# Persistence of Immune Response Elicited by Three Doses of mRNA Vaccine against SARS-CoV-2 in a Cohort of Patients with Solid Tumors: A One-Year Follow-Up

**DOI:** 10.3390/ijms24076731

**Published:** 2023-04-04

**Authors:** Angioletta Lasagna, Irene Cassaniti, Francesca Arena, Federica Bergami, Elena Percivalle, Giuditta Comolli, Antonella Sarasini, Alessandro Ferrari, Daniela Cicognini, Roberta Schiavo, Giuliana Lo Cascio, Paolo Pedrazzoli, Fausto Baldanti

**Affiliations:** 1 Medical Oncology Unit, Fondazione IRCCS Policlinico San Matteo, 27100 Pavia, Italy; 2 Microbiology and Virology Department, Fondazione IRCCS Policlinico San Matteo, 27100 Pavia, Italy; 3 Microbiology Unit, Hospital Guglielmo da Saliceto, 29121 Piacenza, Italy; 4 Department of Internal Medicine and Medical Therapy, University of Pavia, 27100 Pavia, Italy; 5 Department of Clinical, Surgical, Diagnostics and Pediatric Sciences, University of Pavia, 27100 Pavia, Italy

**Keywords:** third dose, BNT162b2 ant-SARS-CoV-2 vaccine, cancer, neutralizing antibody, VOCs, Omicron 5

## Abstract

The role and durability of the immunogenicity of the BNT162b2 mRNA vaccine against severe acute respiratory virus 2 (SARS-CoV-2), in cancer patients one year after receiving the third dose have to be elucidated. We have prospectively evaluated the long-term immunogenicity of the third dose of the SARS-CoV-2 BNT162b2 mRNA vaccine in 55 patients undergoing active treatment. Neutralizing antibody (NT Ab) titers against Omicron variants and total anti-trimeric S IgG levels were measured one year after the third dose. Heparinized whole-blood samples were used for the assessment of the SARS-CoV-2 interferon-γ release assay (IGRA). Thirty-seven patients (67.3%) showed positive total anti-trimeric S IgG one year after the third dose. Looking at the T-cell response against the spike protein, the frequency of responder patients did not decrease significantly between six and twelve months after the third dose. Finally, less than 20% of cancer patients showed an undetectable NT Ab titer against BA.1 and BA.5 variants of concern (VOCs). Underlying therapies seem to not affect the magnitude or frequency of the immune response. Our work underlines the persistence of humoral and cellular immune responses against BNT162b2 in a cohort of cancer patients one year after receiving the third dose, regardless of the type of underlying therapy.

## 1. Introduction

Beta-coronaviruses are commonly associated with human diseases, and, in the last twenty years, two outbreaks of severe respiratory diseases occurred in 2012 and 2003, caused by the Middle East Respiratory syndrome coronavirus (MERS-CoV) and severe acute respiratory syndrome coronavirus (SARS-CoV-1), respectively [1]. A new beta-coronavirus, named severe acute respiratory virus 2 (SARS-CoV-2), the causative agent of coronavirus infectious disease 2019 (COVID-19), was identified in late 2019 in Wuhan (Hubei Province, China) and rapidly spread all over the world in the next few months, leading the World Health Organization to declare a pandemic on 11 March 2020 [2]. In response to the viral pandemic, several vaccines have been proposed, including novel mRNA vaccines, which have a high efficacy of over 90% [3,4]. To date, vaccination represents the key preventative strategy against SARS-CoV-2, but it might be associated with a suboptimal immune response in immunocompromised subjects, including cancer patients, in comparison to the general population [5,6]. Both B- and T-cell-mediated immune responses are elicited by vaccination and involve different mechanisms, including the production of neutralizing antibodies and the elimination of infected cells. So far, neutralizing antibodies are able to prevent viral entry, while the cellular response can prevent severe infection. T-cell subsets perform discrete roles in immunity: in particular, T-cell epitopes, which are less susceptible to antigenic drift, can induce longer-term protection against different SARS-CoV-2 variants [7,8,9].

The highly transmissible SARS-CoV-2 variants of concern (VOCs), such as Omicron variants, have highlighted the need to improve the immune responses induced by vaccines by administering repeated booster doses [10]. Omicron rapidly became the dominant strain [11], and the subsequent Omicron sublineages appear much more transmissible [12]. The increasing infectivity of the original Omicron variant is attributed to accumulated mutations that enhance the binding of the Omicron spike protein to human angiotensin-converting enzyme 2 (ACE2) [13]. The large number of mutations in its spike protein explains the significant antibody evasion of SARS-CoV-2 Omicron [14]. The Advisory Committee on Immunization Practices of the CDC has recommended a COVID-19 vaccine booster be administered to high-risk groups, including patients with cancer, to deal with the potential decline in immunity against SARS-CoV-2 variants [15]. Additionally, real-world evidence of the higher effectiveness of a fourth vaccine dose has been largely provided [16] in different categories, including healthcare workers [17] and transplanted patients [18].

In our previous studies, we investigated the immunogenicity of the third dose of the vaccine in triggering both humoral and cell-mediated immune responses in cancer patients on active treatment immediately after vaccination [19] and six months after the third dose [20]. Limited longitudinal data exist about immune responses one year after three doses of the vaccine, especially in patients with cancer. In this prospective study, we followed immunized cancer patients to evaluate their humoral and cell-mediated responses one year after three doses of the BNT162b2 mRNA anti-SARS-CoV-2 vaccine. We aimed to evaluate the rate of SARS-CoV-2 responder patients 12 months after receiving the third dose and to verify whether an additional fourth dose further improves the immune response.

## 2. Results

### 2.1. Patients’ Characteristics

The original study cohort [19] consisted of 142 patients with solid tumors on active treatment (56 females and 86 males; median age 66 years, range 26–88). The majority of them (76.7%) had lung cancer and received only immunotherapy (83%). Six months after the third dose [20], we analyzed the data of the 83 patients who were still on active treatment (36 females and 47 males; median age 63, range 26–87 years). The current study included 55 patients with solid tumors (27 females and 28 males; median age 61 years, range 26–87 years) who were on active treatment 12 months after the third dose. The third dose was administered at a median of 155 days after the second dose (range 70–190 days). Thirty-three (60%) had lung cancer, ten (18%) had melanoma, six (10%) had breast cancer and the remaining six patients had kidney cancer (4%), head and neck cancer (4%) and gastrointestinal cancer (4%). Based on the type of therapy administered, the large majority of the patients (32/55; 58%) received only immunotherapy, while 17/55 (31%) received only chemotherapy; in a small number of patients (6/55; 11%), both immunotherapy and chemotherapy were administered (Table 1).

### 2.2. Persistence of SARS-CoV-2 Humoral and Cell-Mediated Immune Responses 12 Months after Third BNT162b2 Dose

**Figure 1:**
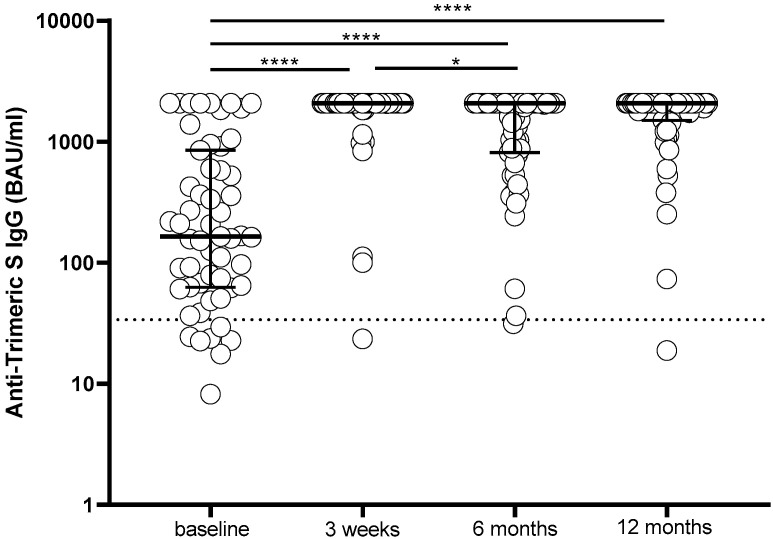
Anti-trimeric S IgG levels elicited in 55 patients by the third dose of the BNT162b2 vaccine measured at the time of administration of the third dose (baseline) and 3 weeks, 6 months and 12 months after the third dose. IgG, immunoglobulin G; S, spike; **** *p* < 0.0001; * *p* < 0.05.

The level of humoral immune response measured by anti-trimeric S IgG was compared at the specified time points in the 55 patients. At the time of the administration of the third dose (T0), 7/55 (12.7%) subjects tested negative for anti-trimeric S IgG, whereas all patients showed a positive serological response three weeks after receiving the third dose (T1), and in 44/55 (80%) patients, it reached the maximum level detectable by our assay (>2080 BAU/mL). Although a decline in the median anti-trimeric IgG response was not observed six months after the third dose (T2), the percentage of subjects showing anti-trimeric S IgG levels higher than 2080 BAU/mL decreased significantly (from 44/55 to 28/55; *p* = 0.0024). Twelve months after receiving the third dose (T3), 37/55 (67.3%) patients showed anti-trimeric S IgG higher than 2800 BAU/mL (Figure 1).

In order to evaluate the role of a fourth dose, anti-trimeric S IgG levels were measured in subjects who received a fourth dose (21/55; 38.2%) at a median of 192 days after the third dose (range 119–222 days) and in subjects who did not receive a fourth dose (34/55; 61.8%). Although 14/21 (66.7%) subjects who received a fourth dose showed the maximum level of response, this frequency was not statistically different from that observed in subjects vaccinated with only three doses (23/34; 67.6%).

Looking at the T-cell response against the spike protein, valid results were obtained in 17 and 41 patients at T2 and T3, respectively. Overall, positive IFN-gamma release after stimulation was observed in 12/17 (70.6%) patients at T2 and in 36/44 (59%) patients at T3 (*p* = 0.4861), suggesting the persistence of a positive response in the large majority of the patients, despite a lower decline in the percentage of responders.

### 2.3. SARS-CoV-2 Neutralizing Response against VOCs One Year after the Third Dose

**Figure 2:**
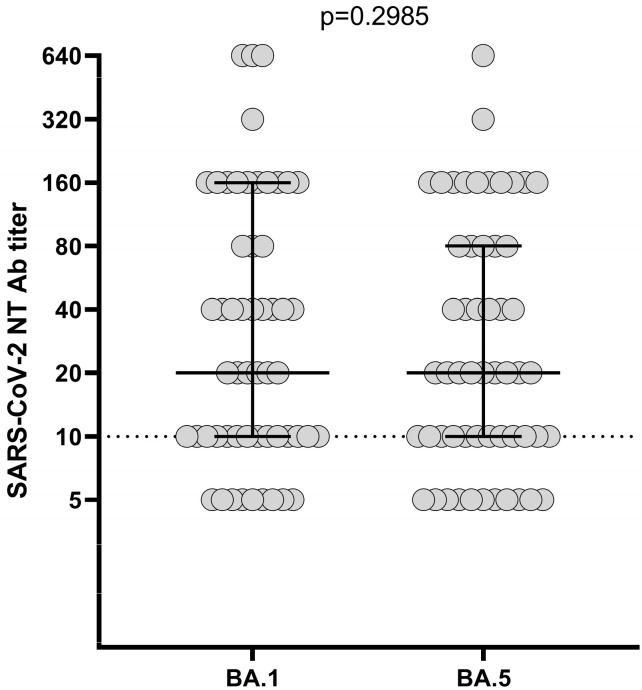
SARS-CoV-2 NT Ab titer measured 12 months after the third dose against Omicron 1 (BA.1) and Omicron 5 (BA.5) variants; NT Ab, neutralizing antibody.

The SARS-CoV-2 NT Ab titer measured 12 months after the third dose was measured against BA.1 and BA.5 VOCs. The median level of the NT Ab titer was not statistically different between the two VOCs (observed against the Omicron variant at T3 (1:20, IQR 1:10–1:160 vs. 1:20, IQR 1:10–1:80; *p* > 0.2985)). Only 9/55 (16.4%) and 11/55 (20%) patients tested negative for BA.1 and BA.5, respectively (Figure 2).

### 2.4. Analysis of Clinical Parameters

**Figure 3:**
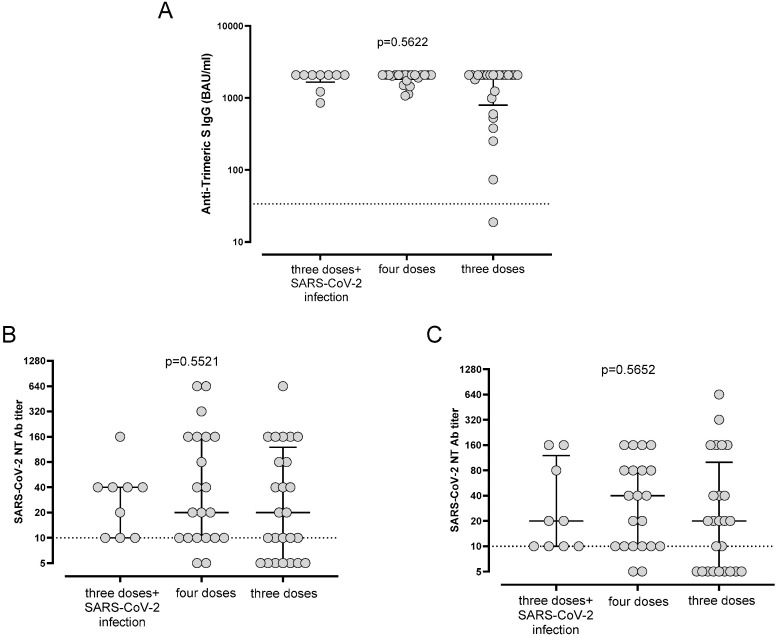
Levels (**A**) of anti-trimeric S IgG and SARS-CoV-2 NT Abs against (**B**) Omicron 1 (BA.1) and (**C**) Omicron 5 (BA.5) variants in patients who had and had not experienced infections at T3. IgG, immunoglobulin G; NT Ab, neutralizing antibody.

No significant differences in terms of the immune response 12 months after the third dose of the vaccine were observed between patients treated with only immunotherapy and patients treated with only chemotherapy or with a combination of immunotherapy and chemotherapy. Similarly, the stage of disease (stage IV vs. stages I to III) did not affect the immune response at 12 months. Overall, 12/55 (21.8%) patients experienced a SARS-CoV-2 infection after the third dose of the vaccine at a median of 184 (range 62–221) days after vaccine administration. Of these, eight patients received antiviral therapy, and only one received monoclonal antibody therapy, while the remaining three patients did not receive any therapy. Of note, only two patients experienced SARS-CoV-2 infections after receiving the fourth dose. None of these patients required hospitalization or oxygen therapy for COVID-19. Overall, S-trimeric IgG levels and SARS-CoV-2 NT Abs against BA.1 and BA.5 were not statistically different between patients who received a fourth dose, patients who received three doses and experienced a SARS-CoV-2 infection, and patients with only three doses of the vaccine in the three groups of patients. However, we observed that the largest number of patients with undetectable NT Abs against BA.1 and BA.5 received only three doses of the vaccine (Figure 3A–C).

## 3. Discussion

It is well known that frail patients, including patients with cancer, have a lower probability of achieving sustained immune responses after vaccination than healthy subjects. Recently, Lee and colleagues, in a UK national COVID-19 cancer cross-sectional study involving patients with cancer after their primary vaccination course (i.e., received at least two doses), demonstrated that 19.2% of patients with hematological cancers did not have a detectable antibody response to the vaccine, compared to 4.2% of patients with solid tumors and 0.1% of healthy subjects [21]. Moreover, they reported that patients with metastatic disease (stage IV) had lower anti-S antibody titers with a consequent higher risk of SARS-CoV-2 breakthrough infection. These results should encourage health professionals to create a more comprehensive strategy to support the pandemic-related needs of the frailest patients [22].

The importance of the booster is well established. In a retrospective population-based cohort study in Ontario, Canada, the authors demonstrated that a third vaccine dose was associated with lower infection rates [23]. Comprehensive knowledge on the duration of BNT162b2 booster dose immunogenicity in cancer patients is crucial for defining the optimal boosting frequency and schedule. Computational modeling can be used to tailor vaccine regimens to high-risk populations. Voutouri and colleagues developed a mathematical model with the known mechanisms of vaccination-induced immunity, SARS-CoV-2 infection and COVID-19 pathophysiology to evaluate the effects of booster doses on the immunogenicity and severity of the clinical course following infection with the ancestral strain and VOCs. The model suggests that a booster of the mRNA vaccine induces a robust enhancement of humoral and cell-mediated immunity and predicts it can provide sufficient protection for more than 12 months in healthy subjects, while in patients with cancer, the booster effect may wane and perhaps should be considered more frequently [24]. In this setting, our study evidenced the persistence of humoral and T-cell-mediated responses elicited by three doses of the mRNA vaccine against SARS-CoV-2 in a cohort of patients with solid tumors on active treatment. In particular, 12 months after receiving the third dose, the large majority of the patients in our cohort showed positive anti-trimeric S IgG results, and in almost 67% of the patients, these results reached the maximum level detectable by our assay. Additionally, although the vaccine used in these patients was not updated for the new variants, the great majority of the patients also showed a sustained response against BA.1 and BA.5 variants.

Several studies have evidenced that Omicron and its sublineages effectively evade two-dose mRNA-vaccine-induced antibodies in healthy individuals with poor neutralization [25,26]. On the other hand, the third mRNA booster dose is able to increase the level of immune response in patients with cancer [27] and the titer of neutralizing antibodies elicited against Omicron variants [28]. Additionally, it has been observed that the effective antibody titer against Omicron persists for at least nine months in the majority of healthy subjects [29].

The potential impact of a breakthrough SARS-CoV-2 infection was also analyzed in our cohort. Although all patients experiencing a SARS-CoV-2 infection after three doses of the vaccine showed a positive NT Ab titer against the two VOCs analyzed, no significant differences in terms of the median level of response were observed in comparison with patients who did not experience a SARS-CoV-2 infection or patients vaccinated with four doses of the BNT162b2 vaccine. In our previous paper [30], we confirmed the benefit of a fourth dose, particularly in terms of enhanced neutralizing activity against the BA.1 variant, with a significant difference between baseline and 3 weeks after the fourth dose. So far, the immunogenicity of the fourth mRNA vaccine dose is in line with previous studies [31].

Although the benefit of a fourth dose in vulnerable subjects is clear, including for other categories of immunocompromised individuals, such as transplanted patients [32], the optimal boosting timing is still uncertain [33]. This issue is important to better define vaccination programs across different risk groups, not only frail patients. In our study, no significant differences in terms of the immune response at 12 months after the third dose of the vaccine were observed according to the oncological treatment. The role of immunotherapy during vaccination is widely debated [34,35,36,37]. Cohen and colleagues have recently evaluated a cohort (n = 42) of patients with solid tumors. They compared patients with three doses of the BNT162b2 vaccine (Pfizer-BioNTech) with patients who received a third BNT162b2 vaccine dose and experienced a breakthrough infection and those who received a fourth BNT162b2 vaccine dose without a breakthrough infection. They demonstrated that the IgG levels of patients with breakthrough infections or with a fourth BNT162b2 vaccine dose were considerably elevated compared to patients with only three doses [38]. Piening and colleagues evaluated whether ICI treatment might influence SARS-CoV-2-specific immune responses in a cohort of 29 vaccinated patients with different types of solid tumors. They reported that the majority of patients had similar antibody titers and T-cell responses before and after immunotherapy and concluded that immunotherapy did not significantly enhance humoral and cell-mediated responses [39].

The strength of our study is the longitudinal tracking of humoral and cell-mediated immune responses in a homogeneous population of patients with solid tumors within a 12-month period. However, this study has several limitations. The small sample size is not sufficient to definitively extend our conclusions about immunogenicity, although this sample size is in line with those in recently published articles [38]. We also recognize that the absence of a control group is another limiting element. Finally, there is a lack of data on the neutralization activities against other current Omicron subvariants such as XBB.

## 4. Materials and Methods

### 4.1. Study Design and Participants

We conducted a prospective, observational cohort study in order to assess humoral and cell-mediated responses after the third dose of the SARS-CoV-2 BNT162b2 vaccine (Pfizer-BioNTech, Mainz, Germany) in patients with cancer. Patients with solid tumors undergoing active anticancer treatment (chemotherapy, immunotherapy or a combination of these types of therapies) were enrolled. A previous SARS-CoV-2 infection was not an exclusion criterion. The patients were referred to the Oncology Units of Fondazione IRCCS Policlinico San Matteo, Pavia. The study (Co-Var) was conducted in accordance with the Strengthening the Reporting of Observational Studies in Epidemiology (STROBE) statement for reporting observational studies [40] and was approved by the local ethics committee (Comitato Etico Area Pavia) and institutional review board. All subjects had provided informed written consent before enrollment.

This is a prospective follow-up report of the primary study. We considered only patients who remained on active treatment 12 months after the third dose of the BNT162b2 anti-SARS-CoV-2 vaccine. The main aim of the study was to evaluate the persistence of the anti-S IgG concentration, and SARS-CoV-2 neutralizing antibodies (NT Abs) against Omicron 1 (BA.1) and 5 (BA.5) variants were evaluated at the end of the follow-up (12 months after the third dose). Additionally, the persistence of the positive spike-specific T-cell response was evaluated in a cohort of patients.

### 4.2. SARS-CoV-2 Variant Isolation and Viral Titration

SARS-CoV-2 strains, including the B.1 strain (carrying the D614G mutation) and Omicron variants (BA.1 and BA.5), were isolated from infected patients’ nasal swabs. Briefly, 200 µL of each sample was inoculated and propagated in the VERO E6 (VERO C1008 (Vero 76, clone E6, Vero E6; ATCC1CRL-1586TM) permissive cell line and titrated to prepare the cell-free virus for the neutralization assay. All strains were sequenced in order to confirm the presence of variant-defining mutations. Complete genome sequencing was performed in order to confirm the presence of variant-defining mutations, and sequences were submitted to GISAID.

### 4.3. Serological Assays

The quantitative characterization of spike-specific IgG antibodies was carried out by performing a Trimeric assay (Liaison, Diasorin, Saluggia, Italy), and the results are given as BAU/mL. Values higher than 33.8 BAU/mL were considered positive. Additionally, NT Abs against the BA.1 and BA.5 strains were measured as previously reported [41]. In detail, 50 µL of the sample, starting from 1:10 in a serial fourfold dilution series, was added to two wells of a flat-bottom tissue culture microtiter plate (COSTAR, Corning Incorporated, New York, NY 14831, USA). In all wells, the same volume of 50 TCID50 of a SARS-CoV2 strain isolated from a symptomatic patient previously titrated was added. The plate was incubated for one hour at 33 °C in 5% CO_2_. All dilutions were made in EMEM with the addition of 1% penicillin, streptomycin and glutammin and 5 γ/mL of trypsin. After 1 h incubation at 33 °C in 5% CO_2_, VERO E6 cells were added to each well and incubated for 48 h at 33 °C in 5% CO_2_. Staining with Gram’s crystal violet solution (Merck KGaA, 64271 Damstadt, Germany) plus 5% formaldehyde 40% *m*/*v* (Carlo ErbaSpA, Arese (MI), Italy) was performed. The blue staining of wells indicated the presence of NT Abs. The neutralizing titer was the maximum dilution with a reduction of 90% of the cytopathic effect (CPE). The results were considered positive when the NT Ab titer was ≥1:10. Positive and negative controls were included in all test runs.

### 4.4. SARS-CoV-2 IFN-g Release Assay

The SARS-CoV-2 cellular response was measured using a specific quantitative interferon-γ release assay (IGRA) in heparinized whole-blood samples, according to the manufacturer’s instructions (Euroimmun, Lübeck, Germany). Briefly, 500 μL of whole blood was added to the stimulator tube coated with the spike antigen. For all samples, negative (unstimulated) and positive control tubes were also added. All samples were mixed gently ten times and incubated overnight at 37 °C in 5% CO_2_. The next day, the samples were centrifuged, and the plasma was stored at −80 °C. IFN-g was detected automatically in the supernatants by an enzyme-linked immunosorbent assay (ELISA, Euroimmun) using the Euroimmun Analyzer I according to the manufacturer’s instructions, and the results are given as mIU/mL. The IFN-g response was defined as spike-stimulated minus unstimulated. Results >200 mIU/mL were considered positive. In the case of an inadequate response to the positive control, the result was considered ‘indeterminate’ and was excluded from the analysis.

### 4.5. Statistical Analysis

Quantitative variables are shown as medians and interquartile ranges (IQRs), while categorical variables are shown as counts and percentages. Comparison between two groups was carried out using the Mann–Whitney (unpaired samples) or Wilcoxon (paired samples) test. Comparison between three or more groups was performed using the Kruskal–Wallis assay. Fisher’s exact test was used for the comparison of categorical variables. All tests were two-tailed, and *p* value < 0.05 was considered significant. All analyses were performed using GraphPad Prism 8.3.0 (GraphPad Software, La Jolla, CA, USA).

## 5. Conclusions

To the best of our knowledge, this is the first report focused on the quantitative evaluation of humoral responses one year after three doses of the SARS-CoV-2 BNT162b2 vaccine. Moreover, we conducted the simultaneous detection of both humoral and cellular immune responses, including the evaluation of SARS-CoV-2 NT Ab activity against the main circulating VOCs (against BA.1 and/or BA.5). This study has some important limitations, including the small sample subset and the lack of a complete follow-up of spike-specific T-cell responses and SARS-CoV-2 NT Abs against the two VOCs BA.1 and BA.5. On the other hand, it provides a comprehensive evaluation of immunogenicity elicited by the SARS-CoV-2 BNT2b2 vaccine in patients with solid cancer on active therapy.

## Figures and Tables

**Table 1:** Demographic and clinical characteristics of enrolled patients.

Sex	Patient No.	Percentage
Female/Male	27/28	49/51
Type of tumor		
Lung cancer	33	60
Melanoma	10	18
Breast cancer	6	10
Kidney cancer	2	4
Head and neck cancer	2	4
Gastrointestinal cancer	2	4
Type of oncological treatment		
ICIs + chemotherapy	6	11
ICIs	32	58
Chemotherapy	17	31
Type of ICI		
Pembrolizumab	20	36
Nivolumab	12	22
Atezolizumab	4	7
Durvalumab	2	4

Legend: ICI: immune-checkpoint inhibitor.

## Data Availability

Data will be provided by the corresponding author upon request.
